# Therapeutic Notes

**Published:** 1896-10-03

**Authors:** 


### THERAPEUTIC NOTES.

### TWO NEW SALTS OF SILVER.

Although not generally known, it appears an undoubted fact that metallic silver has powerful antiseptic properties[1], as indeed, have many other metals; but the latter have been found to act with too much energy on the animal tissue to be of practical use in surgery. Silver, however, in the form of thin rolled foil has in several instances been tried as a dressing for wounds; it is, unfortunately, too clumsy a method for general use; powdered silver, and silver in other forms has been incorporated in the tissues of various forms of gauze and other dressing, and used apparently with some success. The antiseptic properties of this metal appear to be due to the formation of lactate of silver, a salt which is formed by the vital activity of various forms of pathogenic germs, which generate lactic acid during the processes of growth and multiplication. Actual experiment shows that lactate of silver kills the germs of anthrax, staphylococci, and streptococci in five minutes when of the strength of 1 in 1,000, and hinders their development when diluted to 1 in 50,000; if present in the blood in the proportion of 1 in 100,000, it is stated that the germs rapidly perish. With this end in view, lactate of silver, or "actol," as it is called, has been injected subcutaneously; it causes, however, a considerable amount of smarting at the seat of injection, and irritation of the mucous surfaces with which it comes in contact. The citrate of this metal, or "itrol," is free from these inconveniences, and affords an excellent antiseptic powder for various dressings; it consists of a finely-divided powder well adapted for the purposes of insufflation, and it has the advantage over iodoform in that it is perfectly odourless. The citrate or itrol, if used as an insufflation for granulating or unhealthy surfaces, can be used pure or combined with other powders; if used in the form of an ointment the basis can be vaseline, lanoline, or lard, and the strength should be about 1 in 50. In aqueous solution the strength should be about 1 in 4,000, and in this form it can be used for instruments and the irrigation of cavities. For the cleaning of unhealthy surfaces the precaution should be taken of preparing the solution immediately before use to avoid decomposition. For subcutaneous injection the lactate or actol may be employed after preliminary injection, or in combination with cocaine. The dose shou ld be about one-fifth of a grain. Owing to the formation of no insoluble combination in the blood or in the tissue juices, it has the valuable property of maintaining its antiseptic powers for a remarkably long period.

[1] Geschaeftsbes. d. Chem. Fabr. v. Heyden, 1896. [2] Pharm. Cntrlh. xxxvii., 1896, No. 11, pp. 157, 159.

